# Clinical characteristics and laboratory findings of children and adolescents with diabetes

**DOI:** 10.1186/1687-9856-2013-S1-P15

**Published:** 2013-10-03

**Authors:** Se Young Kim, Eun Young Kim, Jin Soon Hwang

**Affiliations:** 1 Bundang Jesaeng General Hospital, Daejin Medical Center, Seongnam, Korea; 2 School of Medicine, Chosun University, Gwangju, Korea; 3 Ajou University School of Medicine, Suwon, Korea

## 

The incidence of childhood type 2 diabetes mellitus (DM) is increasing worldwide in parallel with an increasing prevalence of childhood obesity. We investigated the type of diabetes and the clinical characteristics in the newly diagnosed diabetic children.

Retrospective analysis of clinical characteristics was done in 125 newly diagnosed diabetic children and adolescents under 18 years of age in Korea from March 2003 to December 2010. Children diagnosed with type 1 diabetes were 100 out of 125 (80%) and 25 out of 125 (20%) were type 2 diabetes. Mean age of onset was 9.26 ± 0.99 years and there was no seasonal variation of incidence. 32% of children with type 1 diabetes presented initially with diabetic ketoacidosis. Mean body mass index (BMI) was 16.8 ± 3.8 kg/m^2^, mean blood glucose level was 457.6 ± 212.5 mg/dL and mean glycated hemoglobin (HbA1c) level was 12.1 ± 2.28%. Positive result was revealed in 52% of the subjects with type 1 diabetes for antibodies to glutamic acid decarboxylase (GAD), 3% for islet-cell antibodies (ICA), 25% for insulin autoantibodies (IAA) and 63% showed positive results for at least one of these autoantibodies. 25 patients (20%) were diagnosed with type 2 diabetes. Mean age of onset of type 2 diabetes was 12.2 ± 3.4 years. 12 out of 25 (48%) subjects were diagnosed with type 2 diabetes in the process of evaluating the cause of obesity without any other presenting symptoms. Mean BMI was 28.3 ± 8.7 kg/m^2^, mean blood glucose level was 217.7 ± 105.5 mg/dL and mean HbA1c concentration was 9.0 ± 2.9%. 52% of the subjects diagnosed with type 2 diabetes had a family history of diabetes and 80% were either overweight or obese. Although still not as common as type 1 diabetes among children, type 2 diabetes mellitus increasingly has been seen in children. Routine medical screening in obese children and adolescents or ones with other risk factors of type 2 diabetes should be emphasized to make early diagnosis and start management of type 2 diabetes to improve long-term outcomes.

